# Phenotype-associated microvascular differences in pediatric Behçet’s disease revealed by nailfold videocapillaroscopy

**DOI:** 10.1007/s00431-026-06804-x

**Published:** 2026-02-23

**Authors:** Ufuk Furkan Ozdemir, Elif Kucuk, Lutfiye Koru, Feray Kaya, Zelal Aydin, Serpil Meric Toprak, Betul Aysegul Ayyildiz, Hatice Kubra Dursun, Eda Nur Dizman, Merve Ozen Balci, Kubra Ozturk, Fatih Haslak

**Affiliations:** Department of Pediatric Rheumatology, Istanbul Medeniyet University, Istanbul, Turkey

**Keywords:** Behçet’s syndrome, Nailfold capillaroscopy, Pediatrics, Vasculitis

## Abstract

Behçet’s disease (BD) is a chronic multisystem vasculitis that may begin in childhood. Microvascular dysfunction is central to its pathogenesis, yet pediatric data are scarce. This study evaluated nailfold videocapillaroscopy (NVC) findings in children with BD using standardized methodology and compared microvascular parameters between complete and incomplete disease phenotypes, as well as with age-matched healthy reference data. In this cross-sectional study, pediatric BD patients fulfilling the pediatric Behçet’s disease (PEDBD) criteria underwent NVC following the European Alliance of Associations for Rheumatology (EULAR) microcirculation protocol. Capillary density, morphology, and microhemorrhages were compared between complete and incomplete BD and with age-matched healthy reference data. Associations between NVC parameters and clinical or laboratory findings were analyzed. Complete BD patients (*n* = 15, 40.5%) had significantly lower capillary density and higher apical loop width, tortuosity, dilatation, abnormal capillaries, and microhemorrhage scores than incomplete BD (*n* = 22, 59.5%). Compared with healthy peers, BD patients showed significantly lower capillary density, arterial and venous diameters, and capillary length but higher intercapillary distance and width.

*Conclusion*: Pediatric BD is associated with distinct NVC abnormalities compared with healthy reference data. Differences in capillary density and morphology were observed between complete and incomplete phenotypes. These findings provide a descriptive overview of phenotype-associated microvascular features in pediatric BD.

**Table:** 

**What Is Known:** *• Nailfold videocapillaroscopy (NVC) can detect microvascular abnormalities in adult Behçet’s disease, but pediatric data are extremely limited.* *• Childhood-onset Behçet’s disease often evolves over time from incomplete to complete phenotype, and reliable objective markers reflecting vascular involvement are lacking.* **What Is New:** *• Pediatric Behçet’s disease shows measurable NVC abnormalities compared with healthy children, even in the absence of clinically overt vascular involvement.* *• Microvascular alterations are significantly more pronounced in complete phenotype than incomplete phenotype, suggesting cumulative vascular burden independent of disease activity scores.*

**Supplementary Information:**

The online version contains supplementary material available at 10.1007/s00431-026-06804-x.

## Introduction

Behçet’s disease (BD) is a chronic, relapsing, systemic vasculitis characterized by mucocutaneous, ocular, articular, gastrointestinal, and neurological manifestations. Although the disease predominantly affects young adults, pediatric-onset cases account for approximately 15–20% of all BD patients, often presenting with a more variable clinical course and posing greater diagnostic challenges due to the evolving nature of symptoms over time [1, 2].

Nailfold videocapillaroscopy (NVC) is a noninvasive, accessible tool for in vivo assessment of microvascular structure. Its utility is well established in conditions such as Raynaud’s phenomenon and systemic sclerosis. Recent efforts by the European Alliance of Associations for Rheumatology (EULAR) Study Group on Microcirculation in Rheumatic Diseases have enabled standardization of NVC parameters in pediatric populations [3, 4]. NVC has shown potential in detecting microvascular involvement in BD, particularly in adult cohorts [5, 6]. However, to our knowledge, NVC findings in pediatric BD have not been systematically reported.

This study aimed to evaluate nailfold capillaroscopic features in children with BD using standardized methodology and to explore associations with clinical subtypes and disease activity. Furthermore, we aimed to assess whether NVC could serve as an auxiliary diagnostic tool by evaluating differences from healthy peer.

## Materials and methods

### Study design and patient selection

This cross-sectional study included pediatric patients with BD who attended routine follow-up visits at our tertiary care center between September to November 2025. All patients were evaluated by a pediatric rheumatologist and classified according to the pediatric Behçet’s disease (PEDBD) classification criteria [2]. Patients fulfilling three or more PEDBD items were classified as complete BD, while those fulfilling two items were classified as incomplete BD, in line with previous pediatric literature. This operational distinction reflects early or evolving disease stages rather than a separate diagnostic category and has been previously used to characterize Behçet-like phenotypes in children [7]. During routine clinical visits, we conducted NVC and collected concurrent demographic and clinical data. Laboratory values obtained during these visits, as part of standard care, were extracted from medical records. Patients were excluded if they had comorbid conditions, any condition preventing reliable NVC assessment (e.g., recent nailbed trauma, infection, or cosmetic procedures), or if fewer than four evaluable capillaroscopic images could be obtained.

### Nailfold videocapillaroscopy examination

All patients underwent NVC according to the standardized protocol of the EULAR Study Group on Microcirculation in Rheumatic Diseases [8, 9]. Before examination, participants rested for at least 15 min in a temperature-controlled room (20–22 °C) to ensure peripheral vasomotor stability. The presence of nail polish, cosmetic procedures, nail biting, or periungual trauma was checked using a standardized checklist, and assessments were postponed for 2–4 weeks if any were identified. Capillaroscopy was performed with a Dino-Lite Capillary Scope 200 Pro (MEDL4N Pro) at × 200 magnification. After applying immersion oil, two images were obtained from each of eight fingers (excluding thumbs), yielding 16 images per patient. Only patients with at least four high-quality analyzable images were included. All images were evaluated by a single pediatric rheumatologist with more than 5 years of experience in NVC and formal training in capillaroscopic interpretation, who was blinded to all clinical and laboratory data. The evaluator assessed all images for capillary density, morphology (tortuosity, crossing, dilation, giant capillaries, and abnormal shapes), and microhemorrhages.

Capillary density was defined as the number of capillaries per millimeter in the distal row, with values < 7/mm considered abnormal. Apical loop widths > 20 µm indicated dilatation, while widths ≥ 50 µm were classified as giant capillaries. Capillaries with normal convex or hairpin morphology, including mild tortuosity or crossing, were regarded as normal variants; all others were classified as abnormal. Microhemorrhages were identified as hemosiderin deposits adjacent to the distal row, and their number was recorded [9]. A normal pattern was defined as normal morphology and density (≥ 7/mm) without dilatation or microhemorrhages. A non-specific abnormal pattern was assigned when dilated capillaries, microhemorrhages, or abnormal morphology were present in the absence of scleroderma-specific features. A scleroderma pattern was defined by the presence of giant capillaries or markedly reduced density (≤ 3/mm) in at least one image, following the Fast-Track algorithm [10]. Patients were classified according to the predominant pattern observed across all images.

As no control group was included, NVC findings were compared with reference values previously reported for healthy children in our country assessed using the same standardized protocol [11]. In that reference study, six capillaries (three capillaries per image, from two images) were analyzed from the fourth finger of the non-dominant hand. To ensure methodological consistency, the same approach was applied to our cohort: two images from the non-dominant fourth finger were selected for analysis in each patient. In addition to overall comparisons, age-specific mean values provided in the reference study’s supplementary material were used for year-by-year comparisons between patients and healthy controls.

For methodological clarity, two complementary analytical approaches were applied in this study. For phenotypic comparison, in accordance with the standardized EULAR NVC protocol, all images obtained from each patient (two images from each of eight fingers, totaling 16 images per patient) were evaluated for overall morphological features and for classification of capillaroscopic patterns. Therefore, all comparisons between incomplete and complete BD phenotypes, including the determination of the predominant capillaroscopic pattern and all phenotype-based analyses, were based on the full set of 16 images per patient. For reference comparison, because no contemporaneous healthy control group was included, comparisons between the patient cohort and published healthy pediatric reference data were performed using an identical methodology to that reference study. Accordingly, for these patient–reference comparisons, quantitative measurements were restricted to two images obtained from the non-dominant fourth finger, with three capillaries analyzed per image. This approach was adopted solely to ensure methodological consistency and comparability with the healthy reference dataset.

#### Statistical analysis

All statistical analyses were conducted using IBM SPSS Statistics for Windows, version 22.0 (IBM Corp., Armonk, NY, USA). The Kolmogorov–Smirnov test was used to assess the normality of distribution for continuous variables. Continuous variables were expressed as mean ± standard deviation (SD) for normally distributed data and as median (minimum–maximum) for non-normally distributed data. Categorical variables were presented as numbers and percentages. For comparison of two independent groups, the independent sample *t*-test was applied for normally distributed variables, while the Mann–Whitney *U* test was used for non-normally distributed variables. Categorical variables were compared using the Chi-square test or Fisher’s exact test, as appropriate. To investigate the relationship between capillaroscopic findings and continuous clinical or laboratory variables (e.g., disease duration, acute phase reactants, and disease activity scores), Spearman’s rank correlation analysis was performed. In cases where statistically significant differences were identified through Kruskal–Wallis or chi-square analyses involving more than two groups, appropriate post hoc tests with Bonferroni correction were performed to determine the source of the difference. Since a healthy control group was not included in this study, capillaroscopic measurements were compared to reference values previously reported in healthy children. For this comparison, one-sample *t*-tests were used to evaluate whether the patient data significantly differed from the published age-specific mean values. A two-tailed *p*-value < 0.05 was considered statistically significant for all analyses. Graphical representations and supplementary analyses were performed using GraphPad Prism version 8.0 (GraphPad Software, San Diego, CA, USA).

## Results

### Baseline characteristics

A total of 37 (female, *n* = 22, 59.5%) pediatric patients diagnosed with BD were included in the study. The median age was 16.75 (5.2–17.3) years, and the median disease duration was 52 (14–165) months. While 22 patients (59.5%) were classified as having incomplete BD, 15 patients (40.5%) fulfilled the criteria for complete BD. The most common clinical features were mucocutaneous manifestations (*n* = 30, 81.1%) (oral aphthae, 28; acneiform lesion, 10; pseudofolliculitis, 10; erythema nodosum, 5; genital ulcer, 3; pathergy positivity, 1), and all the patients were under colchicine treatment. Detailed data are summarized in Table 1.

**Table 1:** Baseline characteristics of the patients with pediatric Behçet’s disease

Female gender; (*n*, %)	22 (59.5%)
Current age (years); median (min–max)	16.75 (5.2–17.3)
Disease duration (month); median (min–max)	52 (14–165)
Type of Behçet’s disease	
Incomplete; (*n*, %)	22 (59.5%)
Complete; (n, %)	15 (40.5%)
Clinical findings	
Mucocutaneous manifestations; (*n*, %)	30 (81.1%)
Oral aphthae; (*n*, %)	28 (75.7%)
Genital ulcer; (*n*, %)	3 (8.1%)
Skin lesions; (*n*, %)	17 (45.9%)
*Acneiform lesions; (n, %)*	10 (27%)
*Pseudofolliculitis; (n, %)*	10 (27%)
*Erythema nodosum; (n, %)*	5 (13.5%)
*Positive pathergy test; (n, %)*	1 (2.7%)
Ocular findings; (*n*, %)	5 (13.5%)
Uveitis; (*n*, %)	5 (13.5%)
Retinal vasculitis; (*n*, %)	1 (2.7%)
Vascular involvement; (*n*, %)	2 (5.4%)
Thrombosis; (*n*, %)	2 (5.4%)
Aneurysm; (*n*, %)	1 (2.7%)
Musculoskeletal involvement; (*n*, %)	12 (32.4%)
Arthralgia; (*n*, %)	12 (32.4%)
Gastrointestinal involvement; (*n*, %)	7 (18.9%)
Abdominal pain; (*n*, %)	6 (16.2%)
Diarrhea; (*n*, %)	4 (10.8%)
Constipation; (*n*, %)	5 (13.5%)
Family history	
Behçet’s disease (*n*, %)	16 (43.2%)
Rheumatic disease (*n*, %)	29 (78.4%)
Laboratory findings	
ESR (mm/h); median (min–max)	4 (2–79)
CRP (mg/L); median (min–max)	1.3 (0.06–137.0)
Total leukocyte count (cells/µL); mean ± SD	8359.1 ± 2703.1
Neutrophils (cells/µL); median (min–max)	4230 (1740–14,590)
Lymphocytes (cells/µL); mean ± SD	2659.1 ± 671.9
Monocytes (cells/µL); median (min–max)	530 (200–1180)
Eosinophils (cells/µL); median (min–max)	110 (30–670)
Platelets (cells/µL); mean ± SD	293,459.4 ± 69,183.1
Hemoglobin (g/dL); mean ± SD	13.4 ± 1.4
Treatment	
Colchicine; (*n*, %)	37 (100%)
Prednisolone; (*n*, %)	3 (8.1%)
Azathioprine; (*n*, %)	6 (16.2%)
Cyclophosphamide; (*n*, %)	1 (2.7%)
Adalimumab; (*n*, %)	5 (13.5%)
Disease activity scores	
Pt-GAS; mean ± SD	2.2 ± 2.1
Ph-GAS; median (min–max)	1 (0–10)
PVAS; median (min–max)	2 (0–7)
IBDDAM; median (min–max)	5 (0–45)
BDCAF; median (min–max)	5 (0–10)

*ESR* erythrocyte sedimentation rate, *CRP* C-reactive protein, *Pt-GAS* Patient Global Assessment, *Ph-GAS* Physician Global Assessment, *PVAS* Pediatric Vasculitis Activity Score, *IBDDAM* Iranian Behçet’s Disease Dynamic Activity Measure, *BDCAF* Behçet’s Disease Current Activity Form

### Comparison between incomplete and complete BD groups

**Fig. 1:**
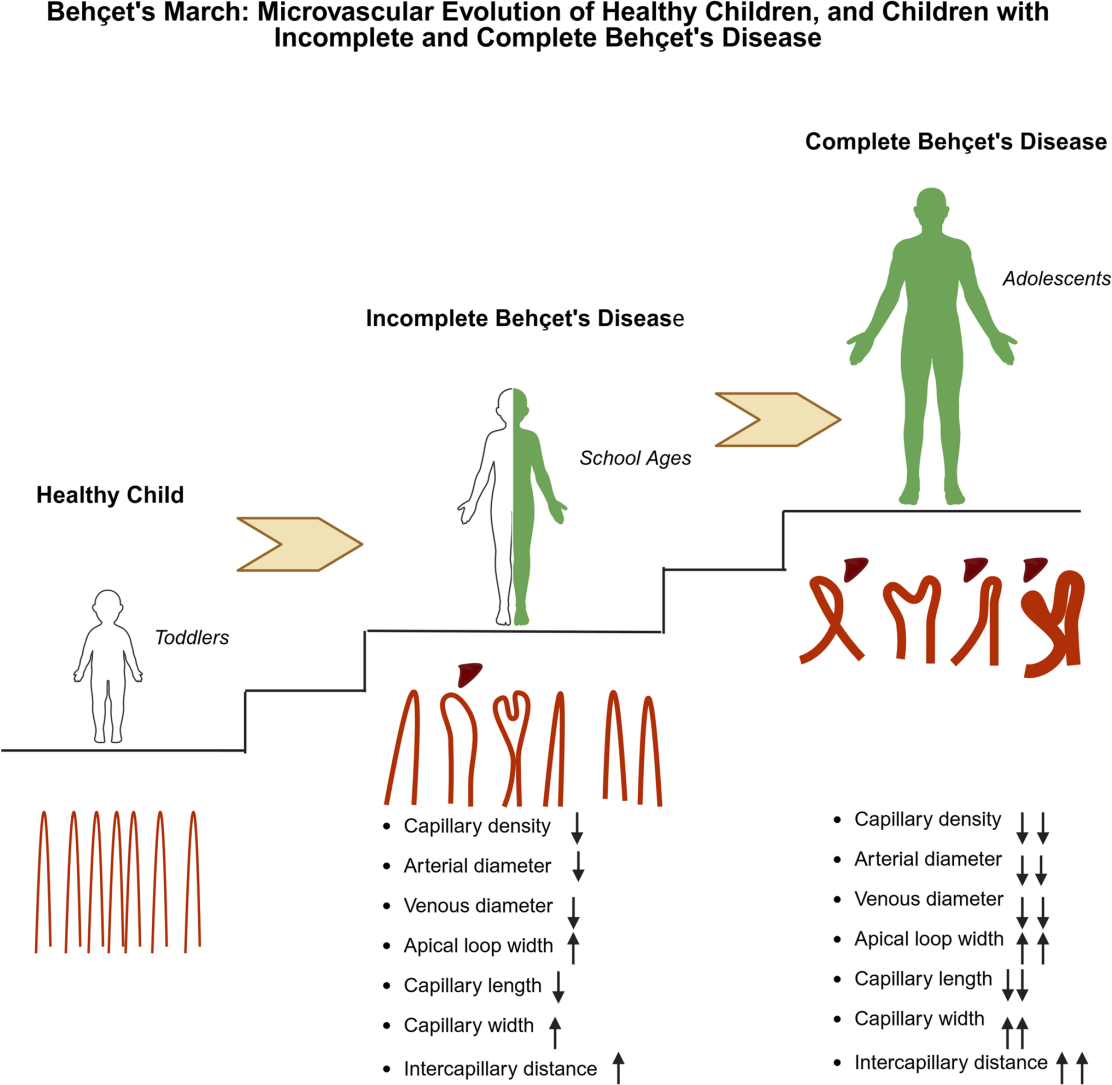
Schematic representation of “Behçet’s March” illustrating the microvascular evolution across disease stages in children

**Fig. 2:**
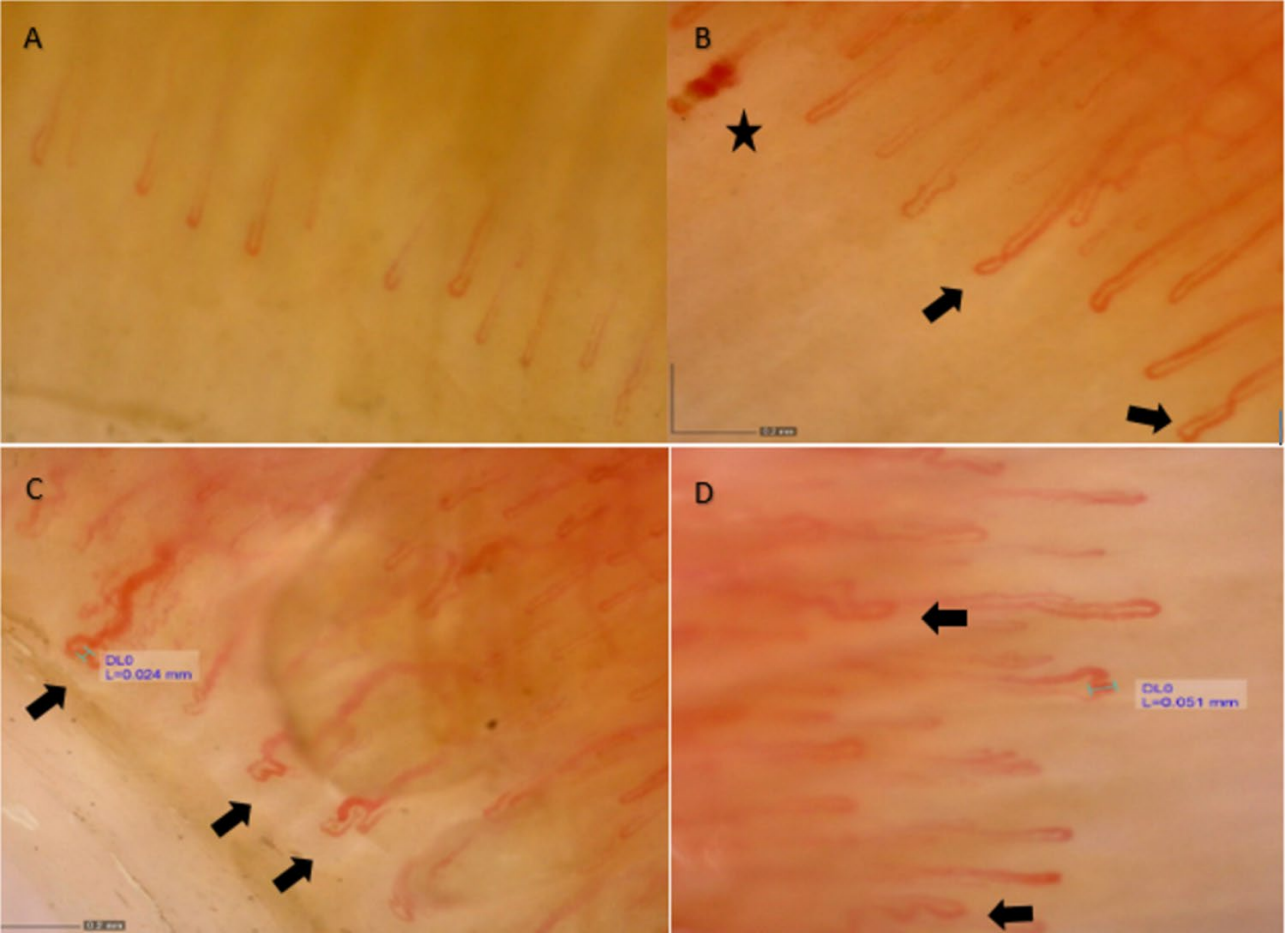
Representative examples of capillaroscopy images from patients with Behçet’s disease (× 200 magnification). **A** Normal capillary morphology with regularly arranged loops and normal diameters. **B** Capillary microhemorrhage: focal dark area due to perivascular hemosiderin deposition following minor capillary leakage (star) and cross capillaries (arrows). **C** Abnormally shaped capillaries deviating from the standard morphology (arrows) and dilated capillary characterized by enlargement of the capillary loop (20–50 μm). **D** Giant capillary presenting with a markedly enlarged diameter (> 50 μm) and capillary tortuosity (arrows)

A reduction in capillary density was observed in all patients with complete BD (100%), compared to only 22.7% of those with incomplete BD (*p* < 0.001). Similarly, the mean capillary density was significantly lower in the complete group than in the incomplete group (6.1 ± 0.4/mm vs. 7.6 ± 0.7/mm, *p* < 0.001). The apical loop width was significantly higher in the complete BD group (22.8 ± 5.8 µm vs. 17.1 ± 2.2 µm, *p* < 0.001). The presence of abnormal capillaries was also common in both groups (complete BD: 93.3% vs. incomplete BD: 95.5%, *p* = 1.000), yet the mean score was significantly higher in the complete BD group (1.18 ± 0.63 vs. 0.67 ± 0.51, *p* = 0.012). Tortuous capillaries were seen in all patients in both groups, with a significantly higher mean score in the complete group (1.85 ± 0.71 vs. 1.37 ± 0.58, *p* = 0.031). The dilated capillary scores (0.59 ± 0.22 vs. 0.23 ± 0.20, *p* < 0.001) and the microhemorrhage scores (0.29 ± 0.11 vs. 0.14 ± 0.18, *p* = 0.007) were significantly higher in the complete group**.** The normal capillary morphology was significantly more common in incomplete BD group (8 (36.4%) vs. 0; *p* = 0.008). Detailed data are available in Table 2 and Fig. 1. Representative nailfold capillaroscopy images from patients with pediatric BD, illustrating common morphological abnormalities such as tortuous, dilated, and giant capillaries, are presented in Fig. 2.

**Table 2:** Comparison of nailfold capillaroscopy findings between incomplete and complete forms of pediatric Behçet disease

	Incomplete Behçet’s disease (*n* = 22)	Complete Behçet’s disease (*n* = 15)	*p* value
Female gender (*n*, %)	13 (59.1%)	9 (60%)	0.956
Age (years); median (min–max)	15.66 (5.2–17.26)	17.19 (12.41–17.38)	0.005
Capillary morphology; (*n*, %)			0.008*
Normal	8 (36.4%)	0 (0%)	
Non-specific	14 (63.6%)	13 (86.7%)	
Scleroderma pattern	0 (0%)	2 (13.3%)	
Decrease in capillary density; (*n*, %)	5 (22.7%)	15 (100%)	< 0.001
Capillary density (per mm); mean ± SD	7.6 ± 0.7	6.1 ± 0.4	< 0.001
Apical loop width (μm); mean ± SD	17.1 ± 2.2	22.8 ± 5.8	< 0.001
Crossing capillaries (per mm); (*n*, %)	22 (100%)	15 (100%)	-
Crossing capillaries score; median (min–max)	1.0 (0.46–2.41)	1.0 (0.53–2.43)	0.914
Abnormal capillaries (per mm); (*n*, %)	21 (95.5%)	14 (93.3%)	1.000
Abnormal capillaries score; mean ± SD	0.67 ± 0.51	1.18 ± 0.63	0.012
Tortuous capillaries (per mm); (*n*, %)	22 (100%)	15 (100%)	-
Tortuous capillaries score; mean ± SD	1.37 ± 0.58	1.85 ± 0.71	0.031
Dilated capillaries (per mm); (*n*, %)	18 (81.8%)	15 (100%)	0.131
Dilated capillaries score; mean ± SD	0.23 ± 0.20	0.59 ± 0.22	< 0.001
Giant capillaries (per mm); (*n*, %)	0 (0%)	2 (13.3%)	0.158
Giant capillaries score; median (min–max)	0 (0–0)	0 (0–0.23)	0.083
Microhemorrhages (per mm); (*n*, %)	16 (72.7%)	15 (100%)	0.063
Microhemorrhages score; mean ± SD	0.14 ± 0.18	0.29 ± 0.11	0.007
Family history of Behçet’s disease; (*n*, %)	10 (45.5%)	6 (40%)	0.742
Family history of inflammatory disease; (*n*, %)	19 (86.4%)	10 (66.7%)	0.228

^*^The significant difference in morphology (*p* = 0.008) was due to the presence of normal morphology only in the incomplete Behçet’s disease group

### Comparison with healthy pediatric reference data

To assess microvascular alterations in pediatric BD, capillaroscopic measurements of our patients were compared with age-matched data from a previously published study on healthy children, using the same standardized methodology [11] (Table 3). In the 8–10 age subgroup, arterial diameter (8.2 ± 0.09 µm vs. 11.5 ± 3.6 µm, *p* = 0.014) and venous diameter (14.1 ± 0.04 µm vs. 14.6 ± 4.2 µm, *p* = 0.041) were found to be significantly lower in BD patients. In the 11–17 age group, which comprised the majority of our cohort, capillary density was significantly lower in BD patients compared to healthy children (7.2 ± 1.3 capillaries/mm vs. 8.7 ± 1.8 capillaries/mm, *p* < 0.001). Arterial diameter (9.6 ± 2.1 µm vs. 12.6 ± 4.3 µm, *p* < 0.001), venous diameter (14.4 ± 2.8 µm vs. 15.8 ± 4.7 µm, *p* = 0.012), and capillary length (264.4 ± 94.2 µm vs. 334.5 ± 108.8 µm, *p* < 0.001) were also significantly reduced in the patient group. In contrast, intercapillary distance was increased in BD patients (148.8 ± 38.3 µm vs. 111.0 ± 57.8 µm, *p* < 0.001), as was capillary width (45.6 ± 9.4 µm vs. 41.6 ± 8.7 µm, *p* = 0.017). Similar differences were observed in the 15–17 age subgroup: Capillary density (7.0 ± 1.3 vs. 9.1 ± 2.0/mm, *p* < 0.001), arterial diameter (9.6 ± 2.3 µm vs. 13.2 ± 4.6 µm, *p* < 0.001), venous diameter (14.5 ± 3.1 µm vs. 16.3 ± 4.9 µm, *p* = 0.007), capillary length (270.5 ± 102.3 µm vs. 341.6 ± 115.9 µm, *p* = 0.001), capillary width (46.2 ± 10.4 vs. 41.8 ± 8.9, *p* = 0.037), and intercapillary distance (148.6 ± 40.9 µm *vs.* 105.3 ± 54.1 µm, *p* < 0.001) all remained significantly different between groups.

**Table 3:** Comparison of nailfold capillaroscopy measurements and morphological features between pediatric Behçet’s disease patients and healthy children

	Group 1(5–7 age) (n=1)			Group 2(8–10 age) (n=2)			Group 3(11–14 age) (n=7)			Group 4(15–17 age) (n=27)		
	Healthy	Patient	p	Healthy	Patient	p	Healthy	Patient	p	Healthy	Patient	p
Capillary density (/mm)	6.9 ± 1.1	7.5	-	7.5 ± 1.2	6.0 ± 1.4	0,374	8.4 ± 1.6	8.0 ± 1.0	0.331	9.1 ± 2.0	7.0 ± 1.3	<0.001
Arterial diameter (µm)	10.6 ± 3.1	9.3	-	11.5 ± 3.6	8.2 ± 0.09	0.014	12.2 ± 4.0	9.6 ± 1.4	0.004	13.2 ± 4.6	9.6 ± 2.3	<0.001
Venous diameter (µm)	13.7 ± 4.4	12.1	-	14.6 ± 4.2	14.1 ± 0.04	0.041	15.4 ± 4.5	14.2 ± 1.3	0.079	16.3 ± 4.9	14.5 ± 3.1	0.007
Loop diameter (µm)	13.8 ± 4.3	14.0	-	16.0 ± 5.1	17.0 ± 2.1	0,626	16.9 ± 5.6	17.6 ± 2.4	0.463	17.7 ± 5.7	18.4 ± 5.6	0.507
Capillary length (µm)	288.4 ± 85.3	203.1	-	289.9 ± 84.7	344.0 ± 62.8	0,438	329.2 ± 103.0	240.5 ± 50.9	0.004	341.6 ± 115.9	270.5 ± 102.3	0.001
Capillary width (µm)	41.7 ± 9.1	35.8	-	40.9 ± 9.6	45.7 ± 9.5	0,603	41.4 ± 8.5	43.6 ± 3.7	0,165	41.8 ± 8.9	46.2 ± 10.4	0.037
Intercapillary distance (µm)	142.0 ± 74.3	167.5	-	134.5 ± 65.2	203.0 ± 8.4	0.056	115.2 ± 60.1	149.8 ± 28.5	0.018	105.3 ± 54.1	148.6 ± 40.9	<0.001
Dilated capillary (%)	5.8%	0%		8.5%	16.6%		8.6%	23.8%		11.4%	27.7%	
Capillary tortuosity <50% (%)	19.6%	100%		15.6%	75.0%		12.4%	78.5%		8.5%	70.3%	
Capillary tortuosity >50% (%)	1.7%	0%		0.8%	0%		0.5%	0%		0%	22.2%	
Capillary crossing <50% (%)	34.9%	50%		36.5%	50%		41.5%	71.4%		42.3%	64.8%	
Capillary crossing <50% (%)	3.1%	0%		3.2%	0%		4.6%	0%		4.4%	5.5%	
Microhemorrhage (%)	3.5%	50%		2.7%	50%		3.0%	21.4%		1.8%	14.8%	
Avascular area (%)	10.4%	50%		7.0%	0%		3.0%	0%		0.4%	0%	

	Total(5–17 age) (n=37)			Pre-pubertal age(5–10 age) (n=3)			Pubertal age(11–17 age) (n=34)					
	Healthy	Patient	p	Healthy	Patient	p	Healthy	Patient	p			
Capillary density (/mm)	8.1 ± 1.7	7.1 ± 1.3	<0.001	7.3 ± 1.2	6.5 ± 1.3	0.405	8.7 ± 1.8	7.2 ± 1.3	<0.001			
Arterial diameter (µm)	11.9 ± 4.0	9.5 ± 2.1	<0.001	11.1 ± 3.4	8.5 ± 0.6	0.020	12.6 ± 4.3	9.6 ± 2.1	<0.001			
Venous diameter (µm)	15.1 ± 4.6	14.3 ± 2.7	0.133	14.2 ± 4.3	13.4 ± 1,1	0.385	15.8 ± 4.7	14.4 ± 2.8	0.012			
Loop diameter (µm)	16.3 ± 5.5	18.0 ± 4.9	0.036	15.2 ± 4.9	16.0 ± 2.2	0.607	17.3 ± 5.7	18.2 ± 5.1	0.280			
Capillary length (µm)	315.0 ± 101.7	267.0 ± 93.2	0.003	289.2 ± 85.0	297.0 ± 92.7	0.897	334.5 ± 108.8	264.4 ± 94.2	<0.001			
Capillary width (µm)	41.5 ± 9.0	45.4 ± 9.3	0.015	41.3 ± 9.4	42.4 ± 8.8	0.845	41.6 ± 8.7	45.6 ± 9.4	0.017			
Intercapillary distance (µm)	122.7 ± 64.7	152.2 ± 38.8	<0.001	138.1 ± 69.3	191.1 ± 21.3	0.050	111.0 ± 57.8	148.8 ± 38.3	<0.001			
Dilated capillary (%)	8.7%	25.6%		7.2%	11.1%		9.8%	26.9%				
Capillary tortuosity <50% (%)	13.7%	72.9%		17.5%	83.3%		10.7%	72.0%				
Capillary tortuosity >50% (%)	0.7%	16.2%		1.2%	0%		0.3%	17.6%				
Capillary crossing <50% (%)	39.2%	64.8%		35.8%	50%		41.8%	66.1%				
Capillary crossing <50% (%)	3.9%	4.0%		3.1%	0%		4.5%	4.4%				
Microhemorrhage (%)	2.7%	18.9%		3.1%	50%		2.5%	16.1%				
Avascular area (%)	4.8%	1.3%		8.6%	16.6%		1.9%	0%				

Among the whole patient cohort, BD patients continued to demonstrate significantly lower capillary density (7.1 ± 1.3 vs. 8.1 ± 1.7/mm, *p* < 0.001), arterial diameter (9.5 ± 2.1 µm vs. 11.9 ± 4.0 µm, *p* < 0.001), and capillary length (267.0 ± 93.2 µm vs. 315.0 ± 101.7 µm, *p* = 0.003) compared to age-matched reference values. Intercapillary distance (152.2 ± 38.8 µm vs. 122.7 ± 64.7 µm, *p* < 0.001), apical loop diameter (18.0 ± 4.9 µm vs. 16.3 ± 5.5 µm, *p* = 0.036), and capillary width (45.4 ± 9.3 µm vs. 41.5 ± 9.0 µm, *p* = 0.015) were also significantly higher in BD patients compared to their healthy peers (Table 3). Age-specific comparisons of mean capillaroscopic values were performed using the reference data reported by age in the supplementary material of the healthy cohort study (Supplementary Table 1).

### Correlation analysis

**Fig. 3:**
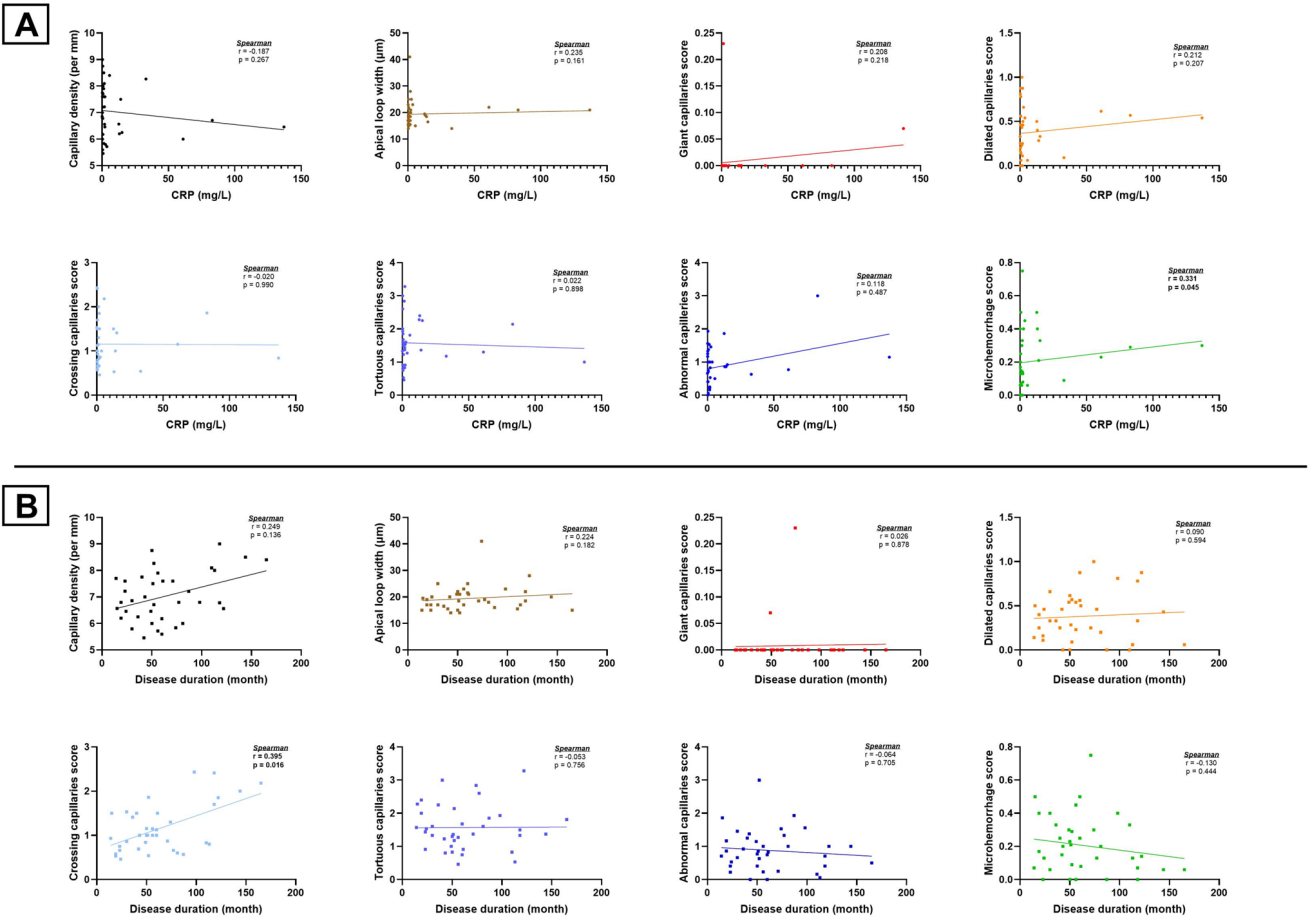
Correlation analyses between capillaroscopic parameters and clinical or laboratory variables in pediatric Behçet’s disease. **A** Scatter plots showing correlations between C-reactive protein levels and capillaroscopic findings. **B** Scatter plots demonstrating correlations between disease duration and the capillaroscopic parameters

A significant positive correlation was observed between disease duration and the crossing capillary score (*r* = 0.395, *p* = 0.016). In addition, microhemorrhage score showed a significant positive correlation with C-reactive protein (CRP) levels (*r* = 0.331, *p* = 0.045). No statistically significant correlations were found between capillaroscopic parameters and any of the disease activity scores, including Behçet’s Disease Current Activity Form (BDCAF), Iranian Behçet’s Disease Dynamic Activity Measure (IBDDAM), Paediatric Vasculitis Activity Score (PVAS), Patient Global Assessment (Pt-GAS), and Physician Global Assessment (Ph-GAS). Correlation analysis between capillaroscopic findings and clinical or laboratory parameters are summarized in Fig. 3.

## Discussion

In this study, we demonstrated that pediatric patients with BD exhibit distinct and measurable microvascular abnormalities in NVC, with more pronounced changes observed particularly in patients with complete BD. Capillary density was markedly reduced in patients with complete BD compared to incomplete cases, and abnormalities including increased apical loop width, dilated, tortuous, and abnormal capillaries, as well as microhemorrhages, were significantly more frequent or severe in this group. Compared with age-matched healthy children, patients with BD exhibited lower capillary density, reduced arterial and venous limb diameters, and shorter capillary length, accompanied by increased capillary width, enlarged apical loop diameter, and consistent alterations in intercapillary distance. These alterations are consistent with measurable microvascular differences in pediatric BD compared with healthy reference data, including in patients without clinically overt vascular involvement.

The differentiation between incomplete and complete BD should be understood in the context of the progression of pediatric disease, rather than as a strict diagnostic dichotomy. In cases of childhood-onset BD, clinical symptoms often develop incrementally, with many patients initially exhibiting limited features before meeting the formal classification criteria. Previous pediatric studies have intentionally employed an incomplete BD category, based on PEDBD criteria, to encompass this early or evolving phase of the disease spectrum [7, 12, 13]. In our cohort, patients with complete BD were significantly older than those with incomplete presentations. This age difference likely reflects the evolving nature of BD, where children initially present with limited features and fulfill classification criteria only over time [14–17]. Such a delay in achieving a complete clinical phenotype underscores the importance of ongoing surveillance in pediatric patients with suggestive symptoms. Additionally, our cohort showed a notably low rate of pathergy positivity. This aligns with the declining diagnostic utility of the pathergy test in pediatric populations, supporting previous findings that it is less reliable in children and varies greatly with ethnicity and technique [18]. Furthermore, it is known that both patients with incomplete BD and those with a short disease duration are less likely to have a positive pathergy test [19, 20]. This finding suggests that the pathergy test has limited diagnostic utility and may not significantly aid in distinguishing between incomplete and complete phenotypes in our cohort.

Although pediatric data on capillaroscopy in BD are not available, evidence from adult cohorts indicates that capillary abnormalities may reflect systemic involvement and inflammatory activity. In adults, abnormal NVC findings most commonly dilated and tortuous capillaries, giant loops, and microhemorrhages have been reported in over half of BD patients [21, 22]. A recent study also noted frequent tortuosity, microhemorrhages, and neoangiogenic changes, though no scleroderma pattern was observed in adults with BD [5]. In our study, similar to adults, more than three-fourth of the children with BD observed to have abnormal morphological findings including dilated and tortuous capillaries and microhemorrhages in NVC.

Our findings indicate that microvascular abnormalities are significantly more prevalent in children with complete BD compared to those with incomplete BD. Complete BD patients demonstrated a marked reduction in capillary density, accompanied by significantly higher scores of tortuous, dilated, and abnormal capillaries, as well as microhemorrhages. Notably, none of the patients with complete BD exhibited normal capillaroscopic morphology, whereas normal patterns were present in one-third of incomplete cases. This between-group difference may be compatible with greater cumulative microvascular alteration in the complete phenotype; however, given the cross-sectional design of the study, these findings should be interpreted as descriptive and hypothesis-generating, and longitudinal studies are required to determine whether NVC changes precede, parallel, or follow clinical phenotype evolution. In line with prior evidence in BD indicating endothelial dysfunction and inflammation-associated vascular injury, the more extensive microvascular involvement observed in complete BD may reflect a higher cumulative vascular burden over time [21]. In this context, a scleroderma pattern was identified in two patients with complete BD according to the Fast-Track algorithm. Importantly, neither of these patients exhibited clinical features or autoantibody profiles suggestive of a scleroderma-spectrum disorder or connective tissue disease overlap. This finding reflects a capillaroscopic pattern classification rather than a clinical diagnosis. In the context of BD, such a pattern may represent advanced or non-specific microvascular damage rather than an underlying scleroderma-spectrum pathology. Nevertheless, the possibility of overlap or misclassification cannot be entirely excluded and should be interpreted with caution, particularly in the absence of longitudinal follow-up.

In our study, there was a positive significant correlation between the disease duration and the crossing capillary score, suggesting progressive microvascular architectural changes over time. Moreover, microhemorrhage scores showed a significant positive correlation with CRP levels; however, this finding should be interpreted cautiously and does not establish a causal link between systemic inflammation and microvascular leakage. There was no association between capillaroscopic findings and standardized disease activity indices in our study. This suggests that NVC abnormalities may not be adequately captured by global disease activity indices in a cross-sectional assessment. Similar selective associations have been reported in adults, where tortuous or dilated capillaries were linked to vascular involvement, earlier disease onset, and thrombotic events features pointing to endothelial dysfunction and hemodynamic stress [5, 21, 22].

In our cohort, capillary density, arterial and venous diameters, and capillary length were significantly lower, whereas capillary width, apical loop diameter, and intercapillary distance were significantly higher than in their healthy peers. These findings align with previous studies comparing adult BD patients and healthy controls. In one cohort, microhemorrhages and crossing capillaries were significantly more frequent in patients with BD-related uveitis than in healthy adults, indicating a link between capillaroscopic abnormalities and disease severity or organ involvement [23]. Similarly, higher rates of capillary dilatation and microhemorrhages in BD patients compared to healthy control group, underscoring the sensitivity of NVC in detecting subtle vascular injury, were reported [6]. However, these findings are not disease-specific and should be interpreted as supportive, descriptive microvascular observations. The presence of comparable microvascular changes in our pediatric BD patients relative to healthy peers further implies that these alterations may develop early, supporting further evaluation of NVC in pediatric BD in prospective studies.

It is important to emphasize that the capillaroscopic abnormalities observed in this cohort are not specific to BD. Unlike the typical scleroderma pattern characterized by early giant capillaries, avascular areas, and late neoangiogenesis seen in systemic sclerosis or Raynaud’s spectrum disorders [9, 10], most patients in our cohort exhibited non-specific abnormalities such as mild dilatation, tortuosity, and microhemorrhages. Similarly, microvascular changes described in antiphospholipid syndrome or mixed connective tissue disease may overlap with these findings [4, 5]. Therefore, NVC findings in pediatric BD should be interpreted as supportive microvascular descriptors rather than diagnostic markers and always in conjunction with clinical and laboratory assessment.

To our knowledge, this study provides the first systematic characterization of NVC findings in a pediatric BD cohort. The study is strengthened by the use of a standardized imaging protocol, comparison against age-matched healthy reference data acquired with the same methodology, and phenotype-based stratification into complete and incomplete BD, which enhances both methodological consistency and clinical interpretability. In addition, exploring associations between NVC measures and laboratory markers as well as disease duration offers a broader, clinically anchored view of microvascular involvement in pediatric BD, while remaining cautious about causal inference. The main limitations include the relatively small sample size, an expected challenge in rare pediatric diseases, which may have reduced power to detect subtle relationships, and the cross-sectional design, which precludes assessment of temporal dynamics or prognostic utility. In addition, patients with complete BD were older and had a longer disease duration than those with incomplete disease, which may have influenced capillaroscopic parameters. Due to the limited sample size, formal adjustment or sensitivity analyses were not performed; therefore, the potential confounding effect of age and disease duration should be considered when interpreting phenotype-based comparisons. Therefore, prospective longitudinal studies are needed to assess whether capillaroscopic alterations evolve over time and correlate with disease activity, organ involvement, or progression from incomplete to complete pediatric BD.

In conclusion, this study demonstrates that children with BD exhibit quantitative NVC differences compared with age-matched healthy reference data, indicating measurable microvascular alterations. These changes were detectable even in patients without clinically overt vascular involvement and were more pronounced in those with complete BD compared with incomplete forms. Overall, these findings describe the spectrum and distribution of NVC abnormalities in pediatric BD within the context of disease phenotype.

## Supplementary Information

Below is the link to the electronic supplementary material.

ESM 1 DOCX (23.6 KB)

## Data Availability

No datasets were generated or analysed during the current study.

## Abbreviations

BD: Behçet’s disease
BDCAF: Behçet’s Disease Current Activity Form
CRP: C-reactive protein
EULAR: European Alliance of Associations for Rheumatology
IBDDAM: Iranian Behçet’s Disease Dynamic Activity Measure
NVC: Nailfold videocapillaroscopy
PEDBD: Pediatric Behçet’s disease
Ph-GAS: Physician Global Assessment
Pt-GAS: Patient Global Assessment
PVAS: Paediatric Vasculitis Activity Score
SD: Standard deviation
