# Correction to: Phenotype-associated microvascular differences in pediatric Behçet’s disease revealed by nailfold videocapillaroscopy

**DOI:** 10.1007/s00431-026-06837-2

**Published:** 2026-03-09

**Authors:** Ufuk Furkan Ozdemir, Elif Kucuk, Lutfiye Koru, Feray Kaya, Zelal Aydin, Serpil Meric Toprak, Betul Aysegul Ayyildiz, Hatice Kubra Dursun, Eda Nur Dizman, Merve Ozen Balci, Kubra Ozturk, Fatih Haslak

**Affiliations:** Department of Pediatric Rheumatology, Istanbul Medeniyet University, Istanbul, Turkey

**Correction to: European Journal of Pediatrics (2026) 185:146**

10.1007/s00431-026-06804-x

The authors of the above-mentioned article wish to correct a typographical error in the **Materials and Methods** section under the "Patient recruitment" subsection.

In the originally published version of this article, the recruitment period was incorrectly stated as "May and August 2025." The correct recruitment period was "**September to November 2025.**"

The authors clarify that ethical approval was obtained prior to the start of recruitment and that this correction is purely a drafting oversight. The study design, data integrity, statistical analyses, and conclusions of the article remain unaffected.

The original article has been corrected.

