# Supplementary material for: Phenotype-associated microvascular differences in pediatric Behçet’s disease revealed by nailfold videocapillaroscopy
Source: Eur J Pediatr. 2026 Feb 23;185(3):146. doi: 10.1007/s00431-026-06804-x (PMC12929361; doi:10.1007/s00431-026-06804-x)
Supplement: Supplementary file 1 — DOCX (23.6 KB) [file 431_2026_6804_MOESM1_ESM.docx]

**Supplementary Table 1.** Comparison of nailfold capillaroscopy measurements between pediatric Behçet disease patients and healthy children according to age groups (5-19) (n=37)*

| Age | Capillary density | | Arterial diameter | | Venous diameter | | Loop diameter | | Capillary width | | Capillary length | | | Intercapillary distance | | |
| --- | --- | --- | --- | --- | --- | --- | --- | --- | --- | --- | --- | --- | --- | --- | --- | --- |
|  | Healthy | Patient | Healthy | Patient | Healthy | Patient | Healthy | Patient | Healthy | Patient | Healthy | Patient | Healthy | | Patient |  |
| 5 | 6.6 ± 1.1 | 7.5 | 10.3 ± 3.2 | 9.3 | 13.1 ± 4.6 | 12.1 | 12.9 ± 4.3 | 14 | 42 ± 9.5 | 35.8 | 278.9 ± 87 | 203.1 | 146.9 ± 76.5 | | 167.5 |  |
| 6 | 6.9 ± 1 | - | 11.1 ± 3 | - | 14.4 ± 4.2 | - | 14.4 ± 3.9 | - | 43.4 ± 9.4 | - | 289 ± 83.7 | - | 139.9 ± 78 | | - |  |
| 7 | 7.5 ± 1.2 | - | 10.3 ± 3 | - | 13.4 ± 4.4 | - | 14 ± 4.6 | - | 39.4 ± 7.9 | - | 296.3 ± 85.1 | - | 136.4 ± 66.3 | | - |  |
| 8 | 7.3 ± 1.1 | 7 | 11.3 ± 3.2 | 8.3 | 14.4 ± 4.1 | 14.1 | 15.4 ± 4.7 | 15.5 | 40.1 ± 8.9 | 39 | 293.5 ± 91.5 | 299.6 | 139.6 ± 62.6 | | 197 |  |
| 9 | 7.7 ± 1.1 | - | 11.6 ± 3.4 | - | 14.8 ± 4.3 | - | 16 ± 5 | - | 42.3 ± 9.9 | - | 290.1 ± 77.4 | - | 129.5 ± 64.8 | | - |  |
| 10 | 7.6 ± 1.3 | 5 | 11.6 ± 3.9 | 8.1 | 14.6 ± 4.3 | 14.1 | 16.6 ± 5.6 | 18.5 | 40.4 ± 9.8 | 52.5 | 286.2 ± 84 | 388.5 | 137.2 ± 66.2 | | 209 |  |
| 11 | 8.3 ± 1.3 | 9.5 | 12.5 ± 4.4 | 10.5 | 15.8 ± 4.9 | 15.8 | 17.7 ± 6.9 | 18.8 | 41.4 ± 7.5 | 47.5 | 322.9 ± 111.2 | 218.6 | 115.7 ± 58.2 | | 139.7 |  |
| 12 | 8.2 ± 1.5 | 8.5 | 12.8 ± 4 | 8.7 ± 0.8 | 16.2 ± 4.5 | 13.0 ± 0.7 | 17.4 ± 5.3 | 19.4 ± 2.2 | 42.8 ± 8.6 | 42.1 ± 2.3 | 331.5 ± 91.7 | 224 ± 17.6 | 122.4 ± 62.7 | | 131.9 ± 40.6 |  |
| 13 | 8.5 ± 1.7 | 7.2 ± 1 | 11 ± 3.3 | 9.7 ± 0.8 | 14.1 ± 4.1 | 14.4 ± 1.2 | 15.7 ± 4.9 | 18.6 | 41 ± 9.2 | 43.1 ± 4.9 | 336.5 ± 115.1 | 289.3 ± 77.4 | 110.5 ± 62.3 | | 144.1 ± 1.2 |  |
| 14 | 8.5 ± 1.7 | 7.5 ± 0.7 | 12.6 ± 4 | 9.9 ± 3 | 15.5 ± 4.3 | 14.6 ± 1.9 | 17.1 ± 5.3 | 14.3 ± 0.4 | 40.6 ± 8.4 | 43.6 ± 5.6 | 325.6 ± 94 | 219.4 ± 50.7 | 112.1 ± 56.7 | | 178.6 ± 28.1 |  |
| 15 | 9.1 ± 2.3 | 6.6 ± 0.9 | 12 ± 3.8 | 10.6 ± 1.8 | 15 ± 4.4 | 13.9 ± 2.1 | 20.2 ± 4.9 | 17.2 ± 1.8 | 40.7 ± 8.1 | 46.8 ± 9 | 314.1 ± 86.1 | 263.5 ± 78 | 110.1 ± 98 | | 157.5 ± 32.7 |  |
| 16 | 9.2 ± 2 | 7.8 ± 1.3 | 14.1 ± 5.5 | 8.5 ± 0.9 | 17.1 ± 5.6 | 13.5 ± 1.1 | 18.9 ± 6.5 | 15.2 ± 3.3 | 41.7 ± 9.7 | 41.6 ± 6.3 | 366.6 ± 127.8 | 253.4 ± 102.6 | 101.6 ± 57.1 | | 129.2 ± 37.8 |  |
| 17 | 9.1 ± 1.6 | 6.9 ± 1.4 | 13.1 ± 4 | 9.6 ± 2.6 | 16.6 ± 4.4 | 14.8 ± 3.6 | 17.7 ± 5.1 | 19.4 ± 6.4 | 42.8 ± 16.2 | 47 ± 11.6 | 337 ± 117.8 | 276.3 ± 112.3 | 105.5 ± 48 | | 150.4 ± 44.1 |  |
| 5-17 (Total) | 8.1 ± 1.7 | 7.1 ± 1.3 | 11.9 ± 4 | 9.5 ± 2.1 | 15.1 ± 4.6 | 14.3 ± 2.7 | 16.3 ± 5.5 | 18 ± 4.9 | 41.5 ± 9 | 45.4 ± 9.3 | 315 ± 101.7 | 267 ± 93.2 | 122.7 ± 64.7 | | 152.2 ± 38.8 |  |
| *The number of patients in each age group: 5 (n=1), 6 (n=0), 7 (n=0), 8 (n=1), 9 (n=0), 10 (n=1), 11 (n=1), 12 (n=2), 13 (n=2), 14 (n=2), 15 (n=5), 16 (n=4), 17 (n=18). | | | | | | | | | | | | | | | | |
|  | | | | | | | | | | | | | | | | |
